# Biomimetic Modeling of Copper Complexes: A Study of Enantioselective Catalytic Oxidation on *D*-(+)-Catechin and *L*-( − )-Epicatechin with Copper Complexes

**DOI:** 10.1155/2008/762029

**Published:** 2008-09-23

**Authors:** Francesco G. Mutti, Roberta Pievo, Maila Sgobba, Michele Gullotti, Laura Santagostini

**Affiliations:** Dipartimento di Chimica Inorganica, Metallorganica e Analitica “Lamberto Malatesta”, Università di Milano, Istituto ISTM-CNR, Via Venezian 21, 20133 Milano, Italy

## Abstract

The biomimetic catalytic oxidations of the dinuclear and trinuclear copper(II) complexes versus two catechols, namely, *D*-(+)-catechin and *L*-( − )-epicatechin to give the corresponding quinones are reported. The unstable quinones were trapped by the nucleophilic reagent, 3-methyl-2-benzothiazolinone hydrazone (MBTH), and have been calculated the molar absorptivities of the different quinones. The catalytic efficiency is moderate, as inferred by kinetic constants, but the complexes exhibit significant enantio-differentiating ability towards the catechols, albeit for the dinuclear complexes, this enantio-differentiating ability is lower. In all cases, the preferred enantiomeric substrate is *D*-(+)-catechin to respect the other catechol, because of the spatial disposition of this substrate.

## 1. INTRODUCTION

Reproducing complex biological reactivity within a simple synthetic molecule is a challenging endeavor with both intellectual and aesthetic goals. The sequence of examining biological reactivity, creating similar chemical architectures, and determining functional reaction conditions for model systems is a process that allows the biological code of reactivity to be deciphered. In the past years, the report on the crystal structures of type 3 copper enzymes (e.g., catechol oxidase, hemocyanins, and tyrosinase) [1–4], as too type 2-type 3 copper enzymes (e.g., ascorbate oxidase, laccase, ceruloplasmin) [5–8] has taken a new turn. The greater availability of such structural information now allows a shift in the role of synthetic modeling from structural and spectroscopic endeavors to the development of functional and catalytic models. Functional models can provide an opportunity to examine a biological reactivity at a small-molecule level of detail through systematic and comparative studies. Although one goal of modeling is reproduction of reactivity, extension of this reactivity beyond the scope of the inspiring system is perhaps an even more important objective. Adequate synthetic models that have similar structural, spectroscopic, and functional properties of active sites of copper proteins are done [9–13]. These models provide many elegant examples of selective and environmentally benign oxidants capable of performing interesting organic transformations, and many of these are copper complexes that use dioxygen as the ultimate oxidant above all in the catecholase activity [14–20]. The interest of our group has mainly focused on dinuclear, and trinuclear [21–28] copper complexes derived from octadentate nitrogen ligands which show catecholase activities. Some of these compounds contain chiral centers [24–28], and we have demonstrated the possibility to induce stereoselectivity in the catalytic oxidation of chiral catechols, such as *L*- and *D*-Dopa and their methyl esters. In the present paper we have extended this investigation on our chiral complexes using as substrates other potential catechols, namely, *D*-(+)-catechin and *L*-(–)-epicatechin, and we have found that the stereoselective catalytic oxidation of these substrates depends on the chirality of the dinuclear or trinuclear copper compounds, and on the spatial disposition of the catechols.

## 2. EXPERIMENTAL

### 2.1. General remarks

**Figure 1:**
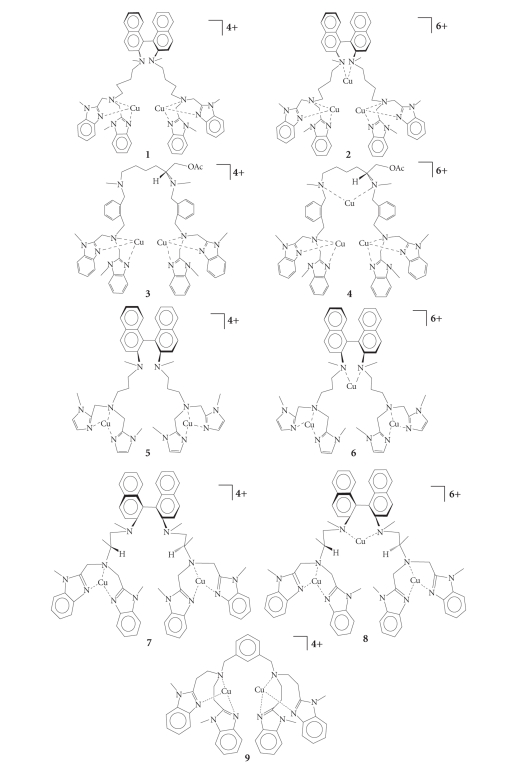
Dinuclear and trinuclear copper complexes used in the biomimetic catalytic oxidations of catechins.

The dinuclear and trinuclear copper(II) complexes [Cu_2_-*R*-DABN-4Bz_4_][ClO_4_]_4_⋅2H_2_O (**1**); [Cu_3_-*R*-DABN-4Bz_4_][ClO_4_]_6_⋅2H_2_O (**2**); [Cu_2_-*L*-Lys-4Bz_4_]⋅[ClO_4_]_4_⋅6H_2_O (**3**); [Cu_3_-*L*-Lys-4Bz_4_][ClO_4_]_6_⋅6H_2_O (**4**); [Cu_2_-*R*-DABN-3Im_4_][ClO_4_]_4_⋅6H_2_O (**5**); [Cu_3_-*R*-DABN-3Im_4_][ClO_4_]_6_⋅2H_2_O (**6**) were prepared as described previously [27]. The trichiral complexes [Cu_2_-*R*-DABN-*L*-Ala-Bz_4_][ClO_4_]_4_ (**7**) and [Cu_3_-*R*-DABN-*L*-Ala-Bz_4_][ClO_4_]_6_ (**8**) were prepared with standard procedures [29]. The dinuclear complex [Cu_2_ *L*-66][ClO_4_]_4_⋅6H_2_O (**9**) was prepared as described previously [30]. All the compounds are shown in Figure 1.

### 2.2. Caution

Although no problems were encountered during the preparation of perchlorate salts, suitable care should be taken when handling such potentially hazardous compounds.

### 2.3. Materials and physical methods

Commercial starting materials were used without purification and the solvents used for the reactions were all spectrophotometric grade. Acetonitrile was distilled from potassium permanganate and sodium carbonate; it was then stored over calcium hydride and distilled before use under nitrogen. The pH of the solutions was measured with an Amel instrument 338. Optical spectra were obtained with HP 8453 diode array spectrophotometer equipped with a thermostated cell holder at the temperature of 20 ± 0.1°C. The data were treated with the commercial program FigSys (BioSoft, Cambridge, UK). Formation kinetics was carried out under pseudo-first-order conditions at 20 ± 0.1°C.

### 2.4. Determination of molar absorptivities of the quinones

It is well known that dinuclear and trinuclear model complexes, like tyrosinase, oxidizes o-diphenols, triphenols, and flavonoids to quinones, but, in all cases, the resulting quinones may undergo nonenzymatic autopolymerization to produce colored compounds. To prevent further reactions of the quinones initially formed, a nucleophilic reagent, 3-methyl-2-benzothiazolinone hydrazone (MBTH), that traps the quinones and generates chromophoric adducts, was used. Unfortunately, no molar absorptivities of these adducts for the substrates were available, so a spectrophotometric method to determine the *λ*_max_ and the molar absorptivities of the adducts was performed. In general, the method is based on the oxidation of the substrate by an excess of sodium periodate, condition under which the reaction was very fast [31, 32]. The unstable quinones were trapped by the nucleophilic reagent (MBTH), and related *λ*_max_ was detected. In all the experiments, only one band developed in the range 300–900 nm, which corresponds to the adducts with MBTH. Based on the recording of *λ*_max_, an experimental design can be carried out to determine the molar absorptivities of the different quinones, for example, performing spectra with different substrate concentrations and fitting the data so obtained to a Lambert-Beer equation by linear regression. Plots of the absorbance values obtained versus the different substrate concentrations allow calculating molar absorptivities. For *D*-(+)-catechin (CQ), the experimental conditions were *λ*_max_ = 459 nm; 50 mM phosphate buffer (pH 7.0)/MeOH (9:1, v:v) at 20 ± 0.1°C; 2 mM NaIO_4_; 1 mM MBTH; substrate concentrations (CQ) from 5 *μ*M to 40 *μ*M; quartz cell 1 cm path length; final volume in cell 2 mL. The coefficient of determination (*r* ^2^) was 0.998 and the molar absorptivity was 17230 M^−1^cm^−1^. For *L*-(−)-epicatechin (EQ), the experimental conditions were *λ*_max_ = 463 nm; 50 mM phosphate buffer (pH 7.0)/MeOH (9:1, v:v) at 20 ± 0.1°C; 2 mM NaIO_4_; 1 mM MBTH; substrate concentrations (EQ) from 5 *μ*M to 45 *μ*M; quartz cell 1 cm path length; final volume in cell 2 mL. The coefficient of determination (*r* ^2^) was 0.994 and the molar absorptivity was 18950 M^−1^ cm^−1^.

### 2.5. Catecholase activities

The kinetics of catalytic oxidation of *D*-(+)-catechin and *L*-(–)-epicatechin were studied by UV-Vis spectroscopy using a magnetically stirred and thermostated 1-cm path length cell. The temperature during the measurements was kept constant at 20 ± 0.1°C. A mixture of aqueous phosphate buffer (50 mM, pH 7.0)-methanol 9:1 (v:v) saturated with atmospheric oxygen was used as solvent. All the kinetic experiments were carried out in duplicate. The experiments performed over a substrate concentration range were initiated by adding a few microlitres of the complexes (final concentrations 0.2–1.4 × 10^−5^ M) to the solution of the substrates; MBTH was maintained 1.0 × 10^−3^ M; the concentration of the substrate was varied between 4.0 × 10^−6^ and 8.0 × 10^−4^ M (final volume 2 mL). The formation of the stable *D*-(+)-catechin-o-quinone-MBTH and *L*-(–)-epicatechin-o-quinone-MBTH adducts was followed through the development of the strong absorption band at 459 nm (*ε* = 17230 M^−1^ cm^−1^) for *D*-(+)-catechin-o-quinone-MBTH, and an absorption band at 463 nm (*ε* = 18950 M^−1^ cm^−1^) for *L*-(–)-epicatechin-o-quinone-MBTH, respectively. In all the experiments, the noise was reduced by reading the absorbance difference between *λ*_max_ and 1100 nm, where the absorption remains negligible during the assay. The initial rates of oxidations were obtained by fitting the absorbance versus time curves in the first seconds of the reactions.

## 3. RESULTS AND DISCUSSION

### 3.1. Stereoselective catalytic oxidations

**Figure 2:**
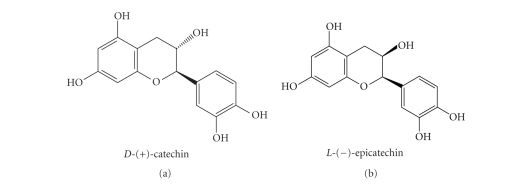
Absolute stereochemistry configuration of the catechols.

The catalytic oxidations of catechols are the most widely employed test reaction to investigate the behavior of tyrosinase and catechol oxidase model complexes. Previous studies [24–27] have shown that chiral dinuclear and trinuclear copper complexes were able to display stereo-discriminating ability towards optically active catechols to give the corresponding o-quinones. To confirm this behavior, new chiral catechols were employed in these catalytic stereoselective oxidations. *D*-(+)-catechin and *L*-(–)-epicatechin (flavan-3-ols) (Figure 2) constitute a class of phenolic compounds ubiquitous in plants and widely found in fruits, vegetables, and beverages [33–35]. In particular, they are one of the major quality factors in grapes and then in the resulting wine [36, 37].

**Scheme 1:**
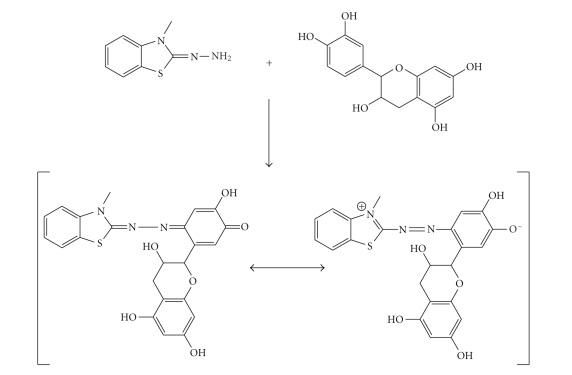
Nucleophilic attach of the reagent MBTH to the catechols.

The catalytic oxidation of polyphenolic substrates, including catechins, was well studied by many authors [38–41]. These reactions take place in the presence of atmospheric oxygen when polyphenol oxidase (PPO) and the corresponding substrates are mixed at the same time. The fundamental first step is the transformation of o-diphenols to the corresponding o-quinones. The fate and stability of o-quinones vary widely, depending both on the phenolic precursor and on environmental factors. In particular, the o-quinones of *D*-(+)-catechin and *L*-(–)-epicatechin were seen to be much less stable than those of other o-quinones. The prolonged autoxidation, either chemical or enzymic, led to the formation of polymers resulting from repeated condensation reactions between an aromatic ring of one molecule with an aromatic ring of another (“head to tail” polymerization mechanism). Depending on how phenolic compounds are oxidized, the condensation products formed from catechins may differ. In fact, the pH of the solution influences considerably the obtained products [42, 43], because at low pH values is favored the formation of colorless condensation products, whereas yellow compounds tended to be formed at higher pH values. To avoid any effect due to pH-dependence of oxidation products and to stop the reaction at quinones formation, a nucleophilic reagent MBTH, that traps the quinones and generates chromophoric adducts, was used (Scheme 1).

We have then studied the pH dependence of the reaction rates and have found that the better pH for the catalytic oxidation of catechins in the presence of the chiral copper complexes is pH 7.0. In order to make a comparison of the catalytic activity among the various chiral complexes reported here, we also studied the catalytic oxidation of the catechins in the presence of the achiral dinuclear complex [Cu_2_(L-66)]^4+^ and MBTH.

Assuming that, for the present biomimetic catalytic reactions, a two-step mechanism of catechol oxidation holds as in the case of our previous studies with dinuclear [22], and trinuclear [23] copper(II) complexes, the following simplified catalytic scheme can be hypothesized, where two molecules of catechol (CatH_2_) per cycle are oxidized to quinone (Q):

(1) $$\begin{array}{l} \text{C}{\text{u}}_{2}^{\text{II}}\;+\;\text{Cat}{\text{H}}_{2}↔\left[\text{C}{\text{u}}_{2}^{\text{II}}/\text{Cat}{\text{H}}_{2}\right]\to \text{C}{\text{u}}_{2}^{\text{I}}\;+\;\text{Q}\;+\;2{\text{H}}^{+},\\ \text{C}{\text{u}}_{2}^{\text{I}}\;+\;\text{Cat}{\text{H}}_{2}\;+\;{\text{O}}_{2}↔\left[\text{C}{\text{u}}_{2}/{\text{O}}_{2}/\text{Cat}{\text{H}}_{2}\right]\to \text{C}{\text{u}}_{2}^{\text{II}}\;+\;\text{Q}\;+\;2{\text{H}}_{2}\text{O}. \end{array}$$

Since, the kinetic experiments showed monophasic behavior and it was impossible to separate the two steps. Thus, either the two steps have a similar rate or the first one is slower. The dependence of the rates of the catalytic reactions as a function of the substrate concentration exhibited a hyperbolic behavior in all cases. However all the complexes exhibited substrate inhibition at high-substrate concentrations, and therefore the kinetic parameters reported in Table 1 were estimated with the equation here reported:

(2) $$v\;=\;\frac{V_{\max}\left[S\right]}{K_{\text{M}}\left(1\;+\;K_{c}{\left[S\right]}^{2}\right)\;+\;\left[S\right]}.$$

The copper complexes with chiral centers exhibited variable degree of stereoselectivity (Table 1) which has been evaluated using kinetic parameters with the following equation:

(3) $$R\%\;=\;\frac{\left[{\left(k_{\text{cat}}/K_{\text{M}}\right)}_{D}\;-\;{\left(k_{\text{cat}}/K_{\text{M}}\right)}_{L}\right]}{\left[{\left(k_{\text{cat}}/K_{\text{M}}\right)}_{D}\;+\;{\left(k_{\text{cat}}/K_{\text{M}}\right)}_{L}\right]}\;\times \;100,$$

where *D* and *L* are *D*-(+)-catechin and *L*-(–)-epicatechin.

**Figure 3:**
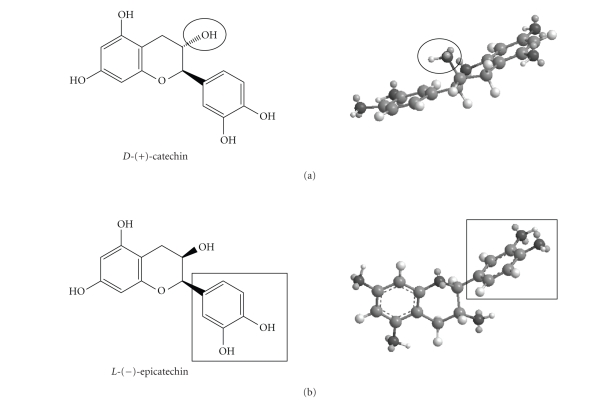
Three-dimensional structure of (a) *D*-(+)-catechin and (b) *L*-(–)-epicatechin with MM2 method.

The catalytic activity of all the complexes, except [Cu_2_(*R*-DABN-4Bz_4_)]^4+^, shows that the preferred coordination of the catechols is for *D*-(+)-catechin. This preference is probably dictated by the chirality of the binaphthyl or lysine residues, as shown by our studies on related complexes [24–26], and especially by the spatial disposition of the catechol substrates. In fact, by simple calculation of molecular energy minimization, *D*-(+)-catechin shows a disposition almost planar with only the hydroxyl group out of plane (Figure 3(a)).

On the contrary, the *L*-(–)-epicatechin shows a more bulky spatial structure, because the two aromatic rings are positioned on orthogonal planes, so the hindrance is very greater than in its isomeric form (Figure 3(b)).

**Scheme 2:**
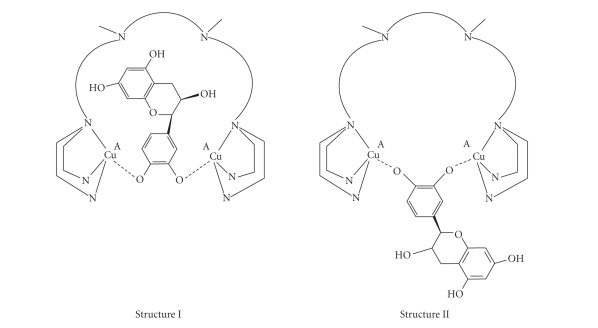
Proposed structures for the putative intermediate adducts formed by the dinuclear copper(II) complexes in the catalytic oxidations of the catechins.

Previous studies on the catalytic oxidations of catechol derivatives demonstrated that the reaction needs the cooperation of two close copper centers [22] to enable the binding of the catechol as a bridging ligand and allow a fast two-electron transfer process. In the dinuclear copper complexes, the catechin substrate can only form a productive complex by binding the catechol residue to the two copper ions in the A sites and that forces the resting part of the molecule to approach the optically active residue so that chiral recognition is possible (Scheme 2).

However, the dinuclear complexes that contain as central core the 1,1-binaphthyl residue show a rigid and bulky structure that reduces the possibility of effective chiral recognition for the catechins. In this case, the coordination of the catechols could occur on the outside of the complexes (Scheme 2, Structure II). The complex [Cu_2_(*L*-Lys-4Bz_4_]^4+^ has a different design. It contains a chiral *L*-Lysine residue as a central unit, which is much more flexible than the 1,1-binaphthyl moiety and this is connected with two arms carrying four benzimidazole donors through a pair of ortho-xylyl spacers. The high flexibility of the spacer and the length of the two arms allow a better chiral recognition of the substrates, as inferred by the kinetic constants and by the degree of stereoselectivity (Scheme 2, Structure I).

**Scheme 3:**
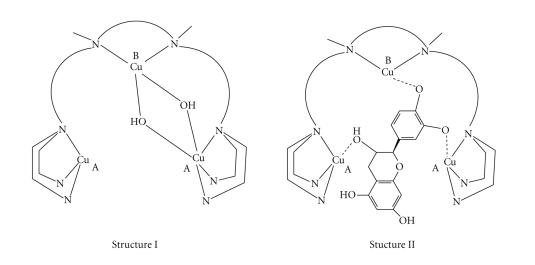
Proposed structures for the trinuclear copper(II) complexes (I) and for the putative intermediate adducts (II) with the catechins.

As reported in the previous papers [26, 27], the trinuclear complexes display a structure in which the Cu(II) center at B site and one of the two centers at A site are mediated by a double hydroxide bridge (Scheme 3, Structure I). In this case, the chiral recognition could depend not only on steric interactions but also by coordination of the free aliphatic hydroxide to the other Cu(II): a site (Scheme 3, Structure II) that allows a significant enantio-differentiating behavior towards optically active substrates. In fact, considering the three-dimensional structures of the two catechins reported before in Figure 3, one notices that the aliphatic hydroxide, in the *D*-(+)-catechin, is opposite to the two catecholic groups, and therefore able to coordinate at the Cu(II) center at A site. The *L*-(–)-epicatechin shows the aliphatic hydroxide too far from the Cu(II) center at A site and, in this case, the interaction needs a modification of the structure of the complexes with a strong tension of the ligands.

For [Cu_2_(L-66)]^4+^, experimental data evidences a very low enantio-differentiation toward *D*-(+)-catechin (see Table 1). This behavior could be due to the stacking interaction between the aromatic ring of m-xylene residue and the aromatic ring far from the catecholic one in the substrate; this interaction should be generated by a parallel disposition of the xylene and the plane of the substrate molecule.

## Figures and Tables

**Table 1:** Kinetic parameters for the stereoselective oxidations of *D*-(+)-catechin and *L*-(–)-epicatechin in methanol-aqueous phosphate buffer, pH 7.0 at 20 ± 0.1°C.

Complexes	*K*_M_ (M)	*k*_cat_ (s^−1^)	*k*_cat_/*K*_M_ (M^−1^ s^−1^)	*R* *%*
Substrate				
[Cu_2_(*R*-DABN-4Bz_4_)]^4+^				
*D*-(+)-catechin	(2.00 ± 0.33) × 10^−5^	(1.21 ± 0.75) × 10^−2^	604	− 32.0
*L*-(–)-epicatechin	(1.20 ± 0.31) × 10^−5^	(1.41 ± 0.96) × 10^−2^	1169	
[Cu_3_(*R*-DABN-4Bz_4_)]^6+^				
*D*-(+)-catechin	(1.33 ± 0.32) × 10^−5^	(1.85 ± 0.12) × 10^−2^	1387	32.8
*L*-(–)-epicatechin	(3.93 ± 0.94) × 10^−5^	(2.75 ± 0.31) × 10^−2^	701	
[Cu_2_(*L*-Lys-4Bz_4_)]^4+^				
*D*-(+)-catechin	(1.14 ± 0.38) × 10^−5^	(1.86 ± 0.18) × 10^−2^	1632	46.0
*L*-(–)-epicatechin	(2.95 ± 0.75) × 10^−5^	(1.78 ± 0.17) × 10^−2^	604	
[Cu_3_(*L*-Lys-4Bz_4_)]^6+^				
*D*-(+)-catechin	(1.37 ± 0.33) × 10^−5^	(2.01 ± 0.02) × 10^−2^	1470	60.4
*L*-(–)-epicatechin	(1.02 ± 0.26) × 10^−4^	(3.71 ± 0.58) × 10^−2^	363	
[Cu_2_(*R*-DABN-3Im_4_)]^4+^				
*D*-(+)-catechin	(2.19 ± 0.70) × 10^−5^	(1.09 ± 0.15) × 10^−2^	498	7.2
*L*-(–)-epicatechin	(1.96 ± 0.58) × 10^−5^	(8.44 ± 0.69) × 10^−3^	431	
[Cu_3_(*R*-DABN-3Im_4_)]^6+^				
*D*-(+)-catechin	(1.01 ± 0.32) × 10^−5^	(5.10 ± 0.39) × 10^−3^	507	42.6
*L*-(–)-epicatechin	(3.51 ± 1.05) × 10^−5^	(7.15 ± 0.80) × 10^−3^	204	
[Cu_2_(*R*-DABN-*L*-Ala-Bz_4_)]^4+^				
*D*-(+)-catechin	(3.60 ± 0.36) × 10^−5^	(2.77 ± 0.11) × 10^−2^	769	8.8
*L*-(–)-epicatechin	(4.81 ± 0.37) × 10^−5^	(3.10 ± 0.11) × 10^−2^	644	
[Cu_3_(*R*-DABN-*L*-Ala-Bz_4_)]^6+^				
*D*-(+)-catechin	(5.05 ± 0.48) × 10^−5^	(6.46 ± 0.15) × 10^−2^	1280	5.3
*L*-(–)-epicatechin	(5.08 ± 0.37) × 10^−5^	(5.84 ± 0.10) × 10^−2^	1150	
[Cu_2_(L66)]^4+^				
*D*-(+)-catechin	(6.69 ± 1.06) × 10^−5^	(7.57 ± 0.60) × 10^−2^	1131	1.9
*L*-(–)-epicatechin	(4.08 ± 0.81) × 10^−5^	(4.44 ± 0.13) × 10^−2^	1088	
